# Mine Tailings Valorization by Electrochemically Stimulated Mineralization from Mildly Acidic Conditions

**DOI:** 10.1021/acssusresmgt.5c00349

**Published:** 2025-12-11

**Authors:** Ivy Wu, Irene E. S. Walker, Robert T. Bell, Kerry C. Rippy

**Affiliations:** † 53405 National Renewable Energy Laboratory, 15013 Denver West Parkway, Golden, Colorado 80401, United States; ‡ 3557 Colorado School of Mines, 1610 Illinois Street, Golden, Colorado 80401-1887, United States

**Keywords:** mineralization, mining, valorization, carbon utilization, electrochemical pH swing

## Abstract

This study presents a high-efficiency electrochemical process for the mineralization of calcium carbonate (CaCO_3_) from mildly acidic mine tailing supernatant water. Electrochemical pH control was used to promote carbonate speciation and precipitation from CO_2_-saturated solutions, achieving >1 mol CaCO_3_ precipitated per mol e^–^ at applied potentials between −1.4 and −1.6 V vs Ag/AgCl. Product morphology and polymorph selectivity were tunable via applied potential, yielding calcite and vaterite phases. Experiments using real mine tailings water confirmed selective calcite precipitation, despite the presence of sulfate and other trace elements. These results highlight a viable route for coupling CO_2_ utilization with mine tailings valorization.

## Graphical abstract

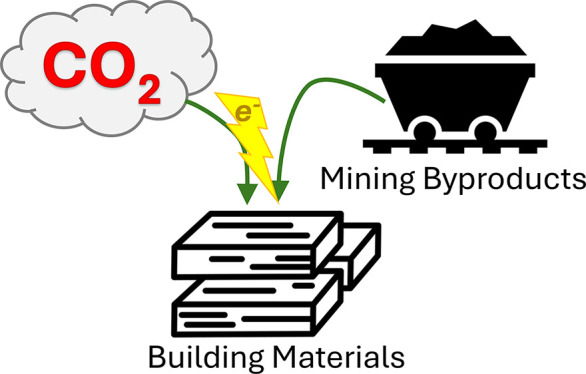

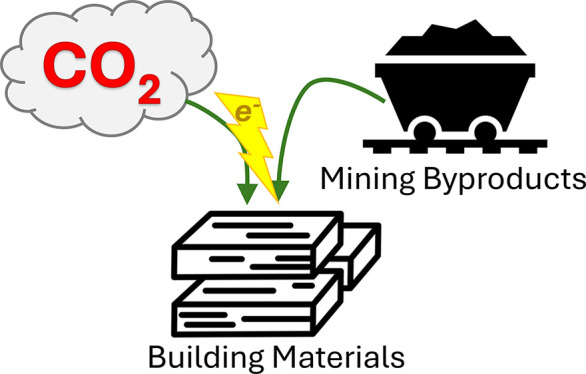

## Introduction

### Background

Mine tailings represent a growing economic and environmental challenge but also a potential resource, covering an estimated global footprint exceeding 200,000 km^2^.^1^ Growing alongside the rising demand for metals, minerals, and other mined materials, tailings are increasingly recognized as a secondary resource for material recovery. Calcium- and magnesium-containing geologic materials are particularly promising feedstocks for carbon mineralization, where CO_2_ reacts with divalent cations to form stable solid carbonates. These solid carbonates are used in several industries, including cement production and could allow for enhanced industrial efficiency and economic savings by repurposing residual material into a raw feedstock.^2–4^

Although the mineralization of CO_2_ is thermodynamically favorable, the kinetics are slow under ambient conditions, requiring mechanical, thermal, or chemical accelerations to achieve practical rates. Walker et al. highlights the different state of the art technologies for mineralization, which include direct gas/solid reactions, direct aqueous reactions, and indirect aqueous processes.^5^ Electrochemical approaches have emerged as an attractive alternative, offering local pH control and carbonate supersaturation near electrode surfaces to drive precipitation without bulk chemical dosing. Chemical reagents for pH-driven precipitation processes can be costly in industrial operations.^6–8^

In electrochemically driven precipitation, hydroxide ions are generated at the cathode via the water reduction reaction (WRR), reaction 1, to promote CO_2_ speciation to carbonate (reactions 2–4), and ultimately yielding CaCO_3_ solids through precipitation with Ca^2+^ (shown as reaction 5, though alternative formation pathways such as reaction with the bicarbonate ion or through hydrated phases are possible^9,10^ ).

1 $${2H}_{2}O+4e^{-}\to H_{2}\left(g\right)+2OH^{-}$$

2 $$CO_{2}\left(aq\right)+H_{2}O↔H_{2}CO_{3}$$

3 $$H_{2}CO_{3}+OH^{-}↔HCO_{3}^{-}+H_{2}O$$

4 $$HCO_{3}^{-}+OH^{-}↔CO_{3}^{2-}+H_{2}O$$

5 $$CO_{3}^{2-}+Ca^{2+}=CaCO_{3}\left(s\right)$$

WRR occurs at more negative potentials than −1.2 V_Ag/AgCl_. In closed batch systems, oxygen is a limiting reactant for the oxygen reduction reaction,^11^ motivating WRR for the cathodic reaction to generate hydroxides. The OH^–^ generated at the cathode results in pH levels much higher near the surface compared to the bulk; e.g., a pH of 10 could form near the cathode even when the bulk solution had a measured pH of 4 ^12,13^ and Lei et al. calculated that local pH values can theoretically reach as high as 13.2 for an assumed maximum local diffusion layer thickness of 1 mm after 1 h of electrolysis.^14^ Figure 1 shows these reactions schematically.

**Figure 1:**
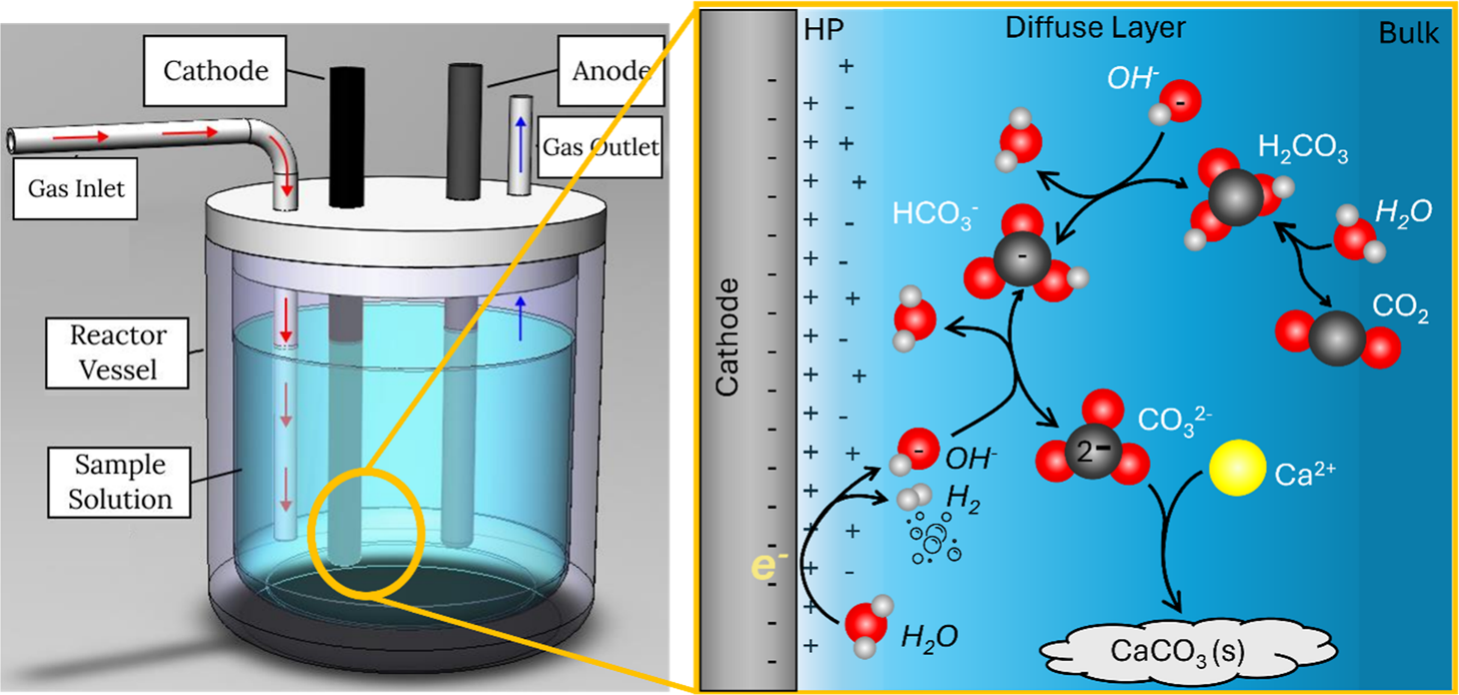
Schematic of electrochemical mineralization cell with relevant electrochemical (black) and chemical (white) reactions.

Previous studies have focused on electrochemically induced CaCO_3_ precipitation for water treatment or carbon capture applications, showing that critical parameters affecting performance include the water chemistry, nature of electrodes, temperature, bulk pH, mass transport, applied current density, or potential.^11,15–17^ For wastewater treatment, studies targeting Ca and Mg removal to avoid scale formation are primarily focused on cathode material properties and reactor design while remaining product agnostic.^18,19^ Additionally, the electrochemical principle of locally generating acids/base has been evaluated for metal removal from acid mine drainage and other wastewater, but with little emphasis on the value of the removed solids.^20,21^ In the context of carbon utilization, the purity and morphology of the carbonate product are critically important.

Many previous studies in the field focused on carbon utilization have investigated electrochemical precipitation processes predominantly under alkaline or neutral conditions, frequently employing seawater, which naturally contains bicarbonate ions that facilitate carbonate precipitation.^22–25^ Devi et al. provided studies on the effect of applied potential, flow rate, and volume of CO_2_ on electrochemical precipitation of calcareous compounds from seawater, finding that the CaCO_3_ and Mg­(OH)_2_ mineral properties can be tuned by altering these parameters and achieving faradaic efficiencies <62% after 72 h of electrolysis.^22^ La Plante et al. developed the “Equatic” process for electrolytic mineral carbonate precipitation from CO_2_ and seawater, finding mixed precipitation of CaCO_3_ and hydrated magnesium carbonates or magnesium hydroxides.^26,27^ Lu et al. developed a 2-mode system for electrochemically stimulated carbonate recovery from simulated seawater brine, achieving 87% product yield of CaCO_3_ after 20 h of electrolysis at −2.5 V_Ag/AgCl_.^28^ The high Mg content in seawater strongly influences the electrochemical precipitation of calcium carbonates.^11^

These systems are less representative of non-alkaline or CO_2_-enriched environments, where bulk pH typically falls near 4 due to the formation of carbonic acid. Additionally, mine tailing supernatants and similar industrial waste streams, while typically circumneutral to alkaline, are chemically complex and may contain trace metals that can alter the mineralization kinetics and phase selectivity. Although Norouzpour et al. reviewed the carbon mineralization methods of industrial waste,^29^ electrochemical pathways remain largely unexplored for these feedstocks. Li et al. developed an electrochemical-based system with a limestone-packed anode to remove valuable metals such as Cu, Cd, and Zn while enriching the effluent with Ca ions, but did not include mineralization with CO_2_.^30^ Expanding electrochemical mineralization to mildly acidic feedstocks of varied compositions, such as mining waters, offers an opportunity to enhance the CO_2_ utilization efficiency and broaden the range of applicable feedstocks. Furthermore, control over carbonate polymorph formation—calcite, aragonite, and vaterite—is a developing area when targeting industrial feedstocks despite these phases exhibiting distinct characteristics and implications for differing product value in cementitious applications.^31–33^

To address these gaps, the present study: (i) presents a pathway for electrochemically stimulated carbonate precipitation from CO_2_-saturated, mildly acidic (pH ≈ 5) solutions; (ii) tunes product morphology and polymorph by applied potential; and (iii) validates the approach using real mine tailing supernatant water, assessing selectivity, purity, and electrode durability.

## Materials and Methods

Calcium chloride dihydrate (99.9%) was obtained from Millipore-Sigma. Stainless steel 316 (250 mesh size) was obtained from McMaster Carr, and Ag/AgCl reference electrode from Pine Instruments. An Autolab Metrohm PGStat302N potentiostat and galvanostat was used for all electrochemical experiments. An Oakton pH5+ probe was calibrated daily and used for all pH measurements.

### Electrochemical Measurements

For synthetic solution studies, 120 mL of a 0.5 M CaCl_2_ solution was poured into a three-necked glass cell with a glass frit (Figure S1), and ultra-high purity bone-dry CO_2_ was bubbled through the frit for 1 h. This process lowers the pH to ∼3.8. The pH was then adjusted to 5.0 by the dropwise addition of 1 M NaOH under vigorous stirring to mimic the initial pH of CO_2_ saturated mine tailings water. For tests with mine tailings supernatants, conditions were identical, except that the pH was not adjusted after bubbling CO_2_, with an initial pH of 5. The system was then conditioned for 30 min, stirring at 60 rpm at open circuit potential (OCP). Stainless steel 316 mesh was used as the working electrode/cathode, with a platinum mesh counter electrode/anode positioned ∼20 mm away and a Ag/AgCl reference electrode positioned close to the working electrode. The chronoamperometry was performed by applying a constant potential for 30 min via a Metrohm USA, Inc Autolab PGStat302N under 60 rpm stirring. The current response was collected during this time, and the bulk pH was measured at a distance 3–4 cm from the cathode. Afterward, the solution was filtered first through a 11 μm Watman filter paper, then through a 0.45 μm Tisch Scientific filter. The electrodes were rinsed with deionized water and then dried at room temperature for 8 h. The mass on the filters and electrodes was weighed and collected. To evaluate electrode changes, electrochemical impedance spectroscopy (EIS) was performed after 30 min of OCP but before chronoamperometry, and immediately after chronoamperometry ended. EIS scans were performed with an AC amplitude of 10 mV over a frequency range of 0.1–1 × 10^6^ Hz.

### Characterization

X-ray diffraction (XRD) was carried out on a Rigaku Ultima XRD instrument from 15° to 60° 2θ (2θ/θ geometry) using copper Κ_α_ radiation. XRD results were analyzed by peak identification and analysis using CrystalDiffract software. Scanning electron microscopy (SEM) and electron-dispersive X-ray spectroscopy (EDS) were performed with an FEI Nova 630 SEM.

## Results and Discussion

### Electrochemically Stimulated Precipitation

The influence of the applied potential will stimulate the WRR. The results of the current response and bulk pH changes to 30 min chronoamperometry at varying constant potentials are shown in Figure 2.

**Figure 2:**
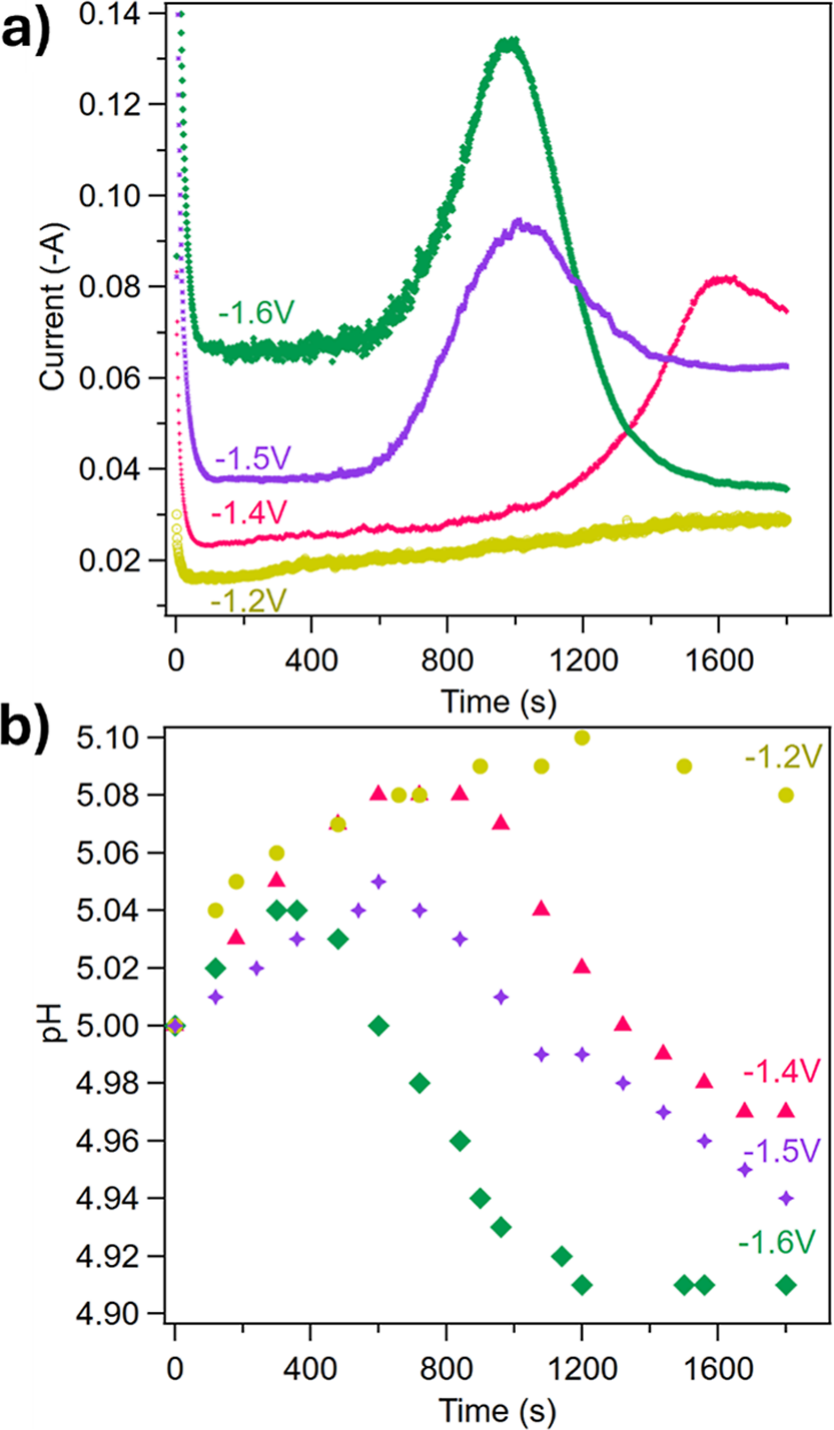
a) Current response and (b) bulk pH during 30 min of chronopotentiometry, −1.2 V (yellow), −1.4 V­(pink), −1.5 V (purple), and −1.6 V (green) vs Ag/AgCl.

The current is relatively stable, with a slow rise in the first 500 s of chronoamperometry at all applied potentials. For potentials more negative than −1.2 V, the current displays a diffusion-limited peak,^34^ followed by stabilization. Increasingly negative voltages correlate with earlier onset and faster decay of this current peak (current increases at 1100, 650, and 650 s of chronoamperometry for applied voltages of −1.4, −1.5, and −1.6 V, respectively). Interestingly, the current stabilizes 20 mA higher at the end of chronoamperometry compared to the beginning (∼65 mA final vs 37 mA initial) for an applied potential of −1.5 V. For −1.6 V, the opposite trend is observed, with the current stabilizing ∼20 mA lower than the initial value after the diffusion-limited peak (35 mA final vs 67 mA initial). Across all tested potentials, the current does not decay to zero, indicating that the electrode surface remains at least partially accessible throughout electrolysis. This behavior, consistent with previous reports, is attributed to the porous nature of CaCO_3_, especially under WRR.^22^ Concurrent gas evolution at the surface of the electrode also aids in removing adhered precipitates.^15,35^

These behaviors indicate that there is a steady induction period prior to precipitation, where the local region near the surface of the working electrode becomes saturated with hydroxides produced via reaction 1, increasing the pH. Figure 1b indeed shows that the bulk pH increases ahead of the rapid current rise, though it is important to note that these are bulk pH measurements that do not fully capture the local pH at the electrode surface. The hydroxides transform dissolved CO_2_ into bicarbonate and carbonate (reactions 2–4). When sufficient carbonates are generated to supersaturate the solution and induce nucleation, CaCO_3_ precipitates. This process shifts reaction 5 to the right while accelerating reaction 1 by consuming OH^–^. Under these conditions, the current rapidly rises until CO_3_^2–^ becomes depleted and the reaction mass transfer limited. Indeed, the conversion of CO_2_ to CO_3_^2–^ was calculated to be ∼40% while the Ca conversion was <5% (Figure S2). Additionally, precipitates on the surface of the electrode increase the resistance to access active sites, further lowering the observed current.

The performance of the electrochemical cell to precipitate carbonate was evaluated at −1.4, −1.5, and −1.6 V and is shown in Figure 3.

**Figure 3:**
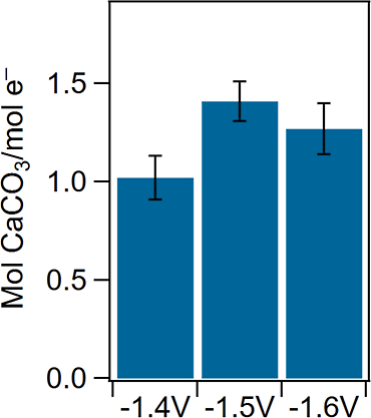
Moles of CaCO_3_ produced per mole of electron passed as a function of applied potential. Error bars are the standard deviation, *n* = 4.

We define the performance as moles of carbonate precipitated normalized by the moles of electrons passed. Because the carbonates are not formed directly from the electrochemical reaction, this metric better encompasses the energy input required for product formation compared to the typically reported metric of current efficiency. Between −1.4 and −1.6 V, >1 mol of product is formed per mole of electron. From the chronoamperometry results, it is likely that the system is limited to CO_2_ as discussed above, and additional overpotential primarily drives water splitting with no additional carbonate formation.

Overall, the high electron to product efficiency is extremely promising, considering that the solution is mildly acidic, which occurs when utilizing concentrated streams of CO_2_. This result highlights the advantages of electrochemically-stimulated mineralization. Fully electrochemically driven growth from an initial pH of 5 would have an efficiency of 50% due to the speciation of CO_2_ to CO_3_^2–^ required for precipitation. That our efficiencies are higher suggests that the solution-driven crystal growth occurs concurrently with electrochemically driven growth once nucleated phases appear. In other words, the advantage of electrochemically driven precipitation is in accelerating the nucleation of carbonates, from which rapid growth is further realized via both electrochemical and solution-driven mechanisms.

### Morphological Properties and Phases of Precipitated CaCO_3_

To further investigate the nature of the precipitates, the precipitates present on the stainless-steel mesh cathode after 30 min of chronoamperometry at −1.4, −1.5, and −1.6 V were imaged by SEM and are shown in Figure 4.

**Figure 4:**
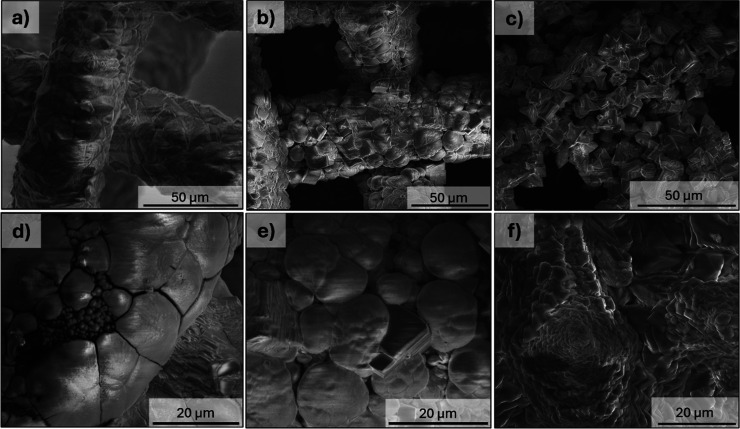
SEM micrographs of precipitates formed after application of 30 min chronoamperometry at (a,d) −1.4 V, (b,e) −1.5 V, and (c,f) −1.6 V vs Ag/AgCl at two different magnifications on the mesh electrode.

From the figure, it is apparent that the morphology of the precipitates is strongly influenced by the applied voltage. At −1.4 V, large polyhedral crystals are observed on the surface of the mesh electrode, appearing partially fused, indicative of extensive crystal growth. Higher magnification images reveal cracks within the deposits, containing smaller rounded precipitates. These cracks are likely due to H_2_ gas evolution, which exfoliates adhered precipitates from the electrode surface. This phenomenon of gas-bubble detachment disrupting surface deposits aligns with the literature^11,15,25^ and applies to all applied potentials investigated in this study. Consistent with this interpretation, Nyquist plots generated at the start and end of chronoamperometry (Figure S3) show a decrease in capacitance and a minor increase in high frequency resistance. The smaller rounded particles within these cracks at −1.4 V may correspond to newly nucleated carbonate phases formed following gas evolution, which have not undergone sufficient growth or ripening to develop into larger particles.^36^ At −1.5 and −1.6 V, the surface of the WE is covered with a mixture of both spherical and cubic crystals. The relatively well-defined nature of these crystals, compared to the agglomerated precipitates at −1.4 V, suggests that higher cathodic potentials increase nucleation rates, leading to rapid generation of smaller carbonate particles in the time frame of the experiment. EDS mapping of the precipitate formed from −1.5 V shows only CaCO_3_ species were precipitated (Figure S4), as expected in the synthetic solution.

To further explore the phases of the precipitates formed at −1.4 V, −1.5 V, and −1.6 V, precipitates were gently scraped from the electrodes after chronoamperometry, and the precipitates were analyzed with XRD (Figure 5). A stacked version of the XRD plot is available in Figure S5.

**Figure 5:**
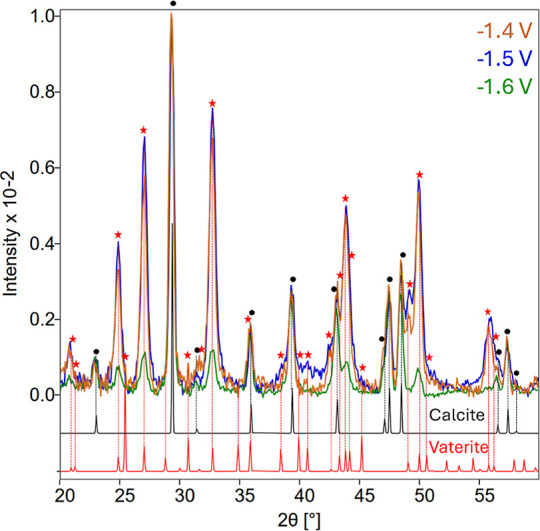
XRD patterns of collected precipitates formed after 30 min of chronoamperometry at −1.4 V (orange), −1.5 V­(purple), and −1.6 V (green), normalized to the highest intensity calcite peak at 29.4° (2θ). Reference peaks for calcite (black lines, black circle) and vaterite (red lines, red star) are shown as reference from ICSD 52151 and ICSD 18127, respectively.

The XRD pattern shows that both calcite and vaterite are present across −1.4 V, −1.5 V, and −1.6 V generated precipitates. Signal intensities are at roughly equivalent levels for −1.4 and −1.5 V, but decreases to a small phase fraction at −1.6 V. This result suggests that increasing the applied potential favors formation of calcite over vaterite. The phase transformation between vaterite and calcite has been shown to be influenced by the Ca^2+^/CO_3_^2–^ ratio, with greater Ca^2+^ ratios favoring calcite formation.^37^ In our system, the local environment is governed by the applied potential, with a sharp current peak corresponding to a mass transfer limited regime at −1.6 V (Figure 2). Therefore, these observations suggest that the rapid generation of OH^–^ at high applied potentials and subsequent precipitation of CaCO_3_ depletes CO_3_^2–^ locally and thus promotes the preferential formation of calcite.

The mineral phases observed are also likely influenced by the duration of exposure to the solution. Precipitates formed from higher applied potentials are exposed to the solution longer than those precipitated from lower potentials due to shortened nucleation times. At higher applied potentials, the enhanced generation of hydroxides promotes local supersaturation near the cathode with respect to carbonate species, ultimately accelerating the nucleation and precipitation of solid carbonates. Given that all experiments in this study were carried out for 30 min, solid carbonates that precipitated faster remained in contact with the electrolyte for a longer portion of the experimental period and had more time to transform into the thermodynamically stable form of calcite. Consistently, Rugabirwa et al. found that vaterite formation was favored when reducing the residence time in CaCO_3_ precipitation.^38^ The ability to control the CaCO_3_ polymorph allows for additional functionalities of the carbonates’ utility in cementitious material, as each polymorph has distinct morphologies, which result in varying final properties.^39^ By controlling the polymorph of the carbonate, we can tune the final mineral to specific applications and increase the value potential for the carbonates.

### Carbonate Precipitation from Mine Tailings Supernatant Water

With the promising results in synthetic solution, we target mineralization from mine tailings supernatant water. Working with our mining partners, we obtained water associated with a tailings pond at a gold–copper mine containing dissolved calcium. This aqueous stream is the decanted water from the tailings pond, which is typically recirculated into the mine operation’s process flow sheet. Table 1 shows the compositions of the major constituents of the tailings water. Trace metals are also present.

**Table 1:** Major Constituents in Mine Tailings Supernatant Water

	mg/L
chloride	2698.45
sodium	1514.75
sulfate	1638.07
calcium	610.48
potassium	231.21
magnesium	56.94
ammonia	20.82
nitrate + nitrite (as N)	2.57

Chronoamperometry was performed, and the current response is shown in Figure 6.

**Figure 6:**
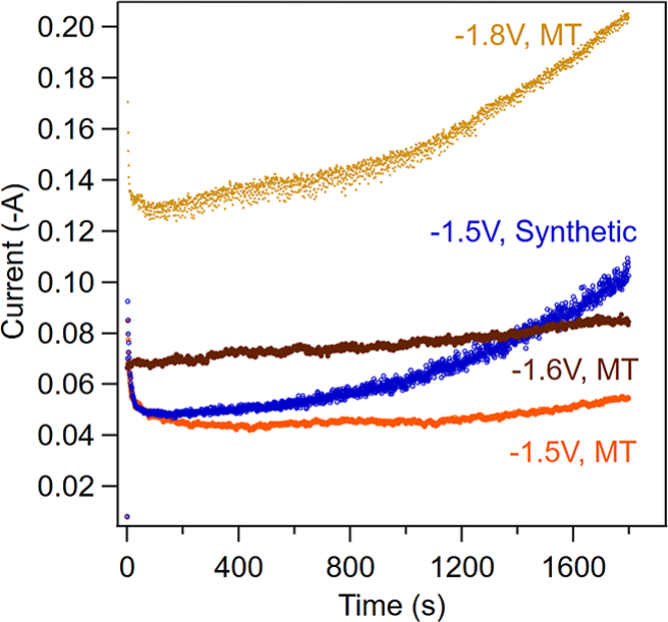
Current response to varying applied potentials in mine tailings supernatant water (MT) and a synthetic solution with comparable Ca concentrations (synthetic). All solutions are CO_2_ saturated with an initial pH of 5.

Electrolysis of real mine tailings supernatant required more negative potentials (>−1.6 V) to induce precipitation, likely attributed to the low solution conductivity of the supernatant and the inhibition of CaCO_3_ nucleation in the presence of sulfate.^40,41^ To assess the phases of the precipitates and possible co-precipitation, XRD, EDS mapping, and SEM were performed for precipitates generated from mine tailings supernatant at −1.8 V, Figure 7.

**Figure 7:**
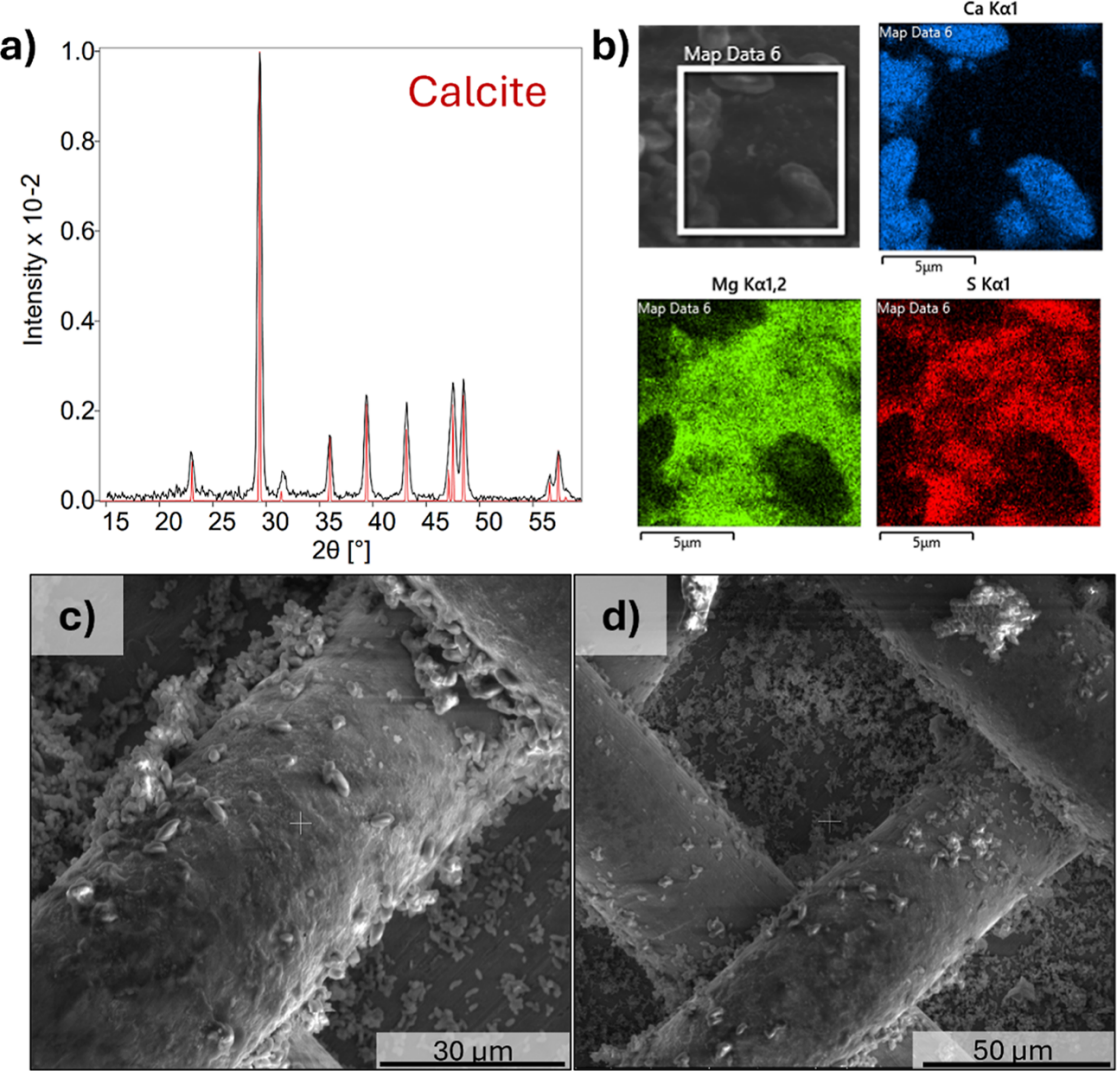
(a) XRD, (b) EDS map, and (c,d) SEM images at two different magnification levels of precipitates on stainless steel mesh formed from mine tailings supernatant water, after 30 min chronoamperometry at −1.8 V vs Ag/AgCl. Red pattern in XRD corresponds to the ICSD 52151 pattern for calcite.

Precipitates formed at −1.8 V exhibit predominantly elongated to rounded morphologies with particle sizes markedly smaller than those obtained from synthetic solutions (Figure 4). XRD analysis confirmed the formation of pure crystalline calcite (ICSD 52151) as the dominant phase (Figure 7a). Notably, no diffraction peaks corresponding to crystalline sulfur-bearing or magnesium-bearing phases were detected; however, the EDS map (Figure 7b) detected both sulfur and magnesium in the precipitates. The sulfur and magnesium are spatially distinct from calcium, suggesting that these phases precipitate as a separate, likely amorphous phase. To investigate the purity of the solids further, the precipitates were digested for ICP-OES analysis and the proportion of each metal calculated, as shown in Table 2.

**Table 2:** % of Metals in Digested Precipitates Formed from Mine Tailings Water

sample ID	% metal
Ca	94.8
Mg	2.5
S	2.1
Na	1.7

These results support the XRD, indicating that calcium carbonate is the predominant solid product formed from the mine tailings water, with minor proportions of magnesium, sulfur, and sodium. Collectively, these findings suggest that in the applied electrochemical conditions, pure calcite is selectively precipitated from mine tailings supernatant water with minimal co-precipitation of other species.

## Conclusions

This work presents an electrochemically driven approach for selective calcium carbonate mineralization from CO_2_ saturated, non-alkaline streams, with applicability to chemically complex systems. By electrochemically controlling the local pH, carbonate supersaturation and precipitation were achieved without the need of bulk chemical additives. At applied potentials between −1.4 and −1.6 V vs Ag/AgCl in synthetic solutions, precipitation efficiencies exceeded one mole of CaCO_3_ per mole of electron transferred, indicating contributions from both electrochemically induced nucleation and subsequent solution-mediated crystal growth. Adjusting the applied potential provided a tunable parameter for polymorph formation: a higher cathodic bias promoted transformation from metastable vaterite to thermodynamically stable calcite, consistent with local shifts in Ca^2+^/CO_3_^2–^ activity and residence time within the reaction boundary layer. Such electrochemical control of phase evolution presents a pathway to tailor carbonate morphology and phase composition for targeted materials applications.

Electrochemical precipitation using real mine tailings supernatant confirmed selective calcite formation despite the presence of sulfate and other ions known to interfere with carbonate precipitation, underscoring the robustness of the electrochemical approach in chemically complex matrices. Minimal electrode passivation further suggests a favorable operational stability and scalability. Collectively, these findings demonstrate that electrochemically stimulated mineralization under mildly acidic conditions can couple CO_2_ utilization with mine tailings valorization in a single, low-chemical-input process. While this study was limited to batch conditions and correspondingly short time scales where reactant depletion effects are minimal, it establishes the foundation for electrochemical mineralization as a low-chemical-input approach to integrate CO_2_ utilization with mine tailings valorization. Ongoing work will examine the impact of foreign ions on electrochemically driven nucleation and growth. Overall, the insights and efficiency metrics reported here drive the viability for carbon mineralization from industrial feedstocks.

## Supplementary Material



## Notes

Photo of electrochemical cell, conversion rate, Nyquist plots, EDS of precipitates from synthetic solutions, stacked XRD plots, and relative standard deviation for calculations (PDF)

## Abbreviations

CDR: carbon dioxide removal
ORR: oxygen reduction reaction
WRR: water reduction reaction
OCP: open circuit potential
EIS: electrochemical impedance spectroscopy
XRD: X-ray diffraction
SEM: scanning electron microscopy
