# Supplementary material for: Mine Tailings Valorization by Electrochemically Stimulated Mineralization from Mildly Acidic Conditions
Source: ACS Sustain Resour Manag. 2025 Dec 11;3(1):76–83. doi: 10.1021/acssusresmgt.5c00349 (PMC12833859; doi:10.1021/acssusresmgt.5c00349)
Supplement: Supplementary file 1 [file rm5c00349_si_001.pdf]

Supplemental Information for

Mine Tailings Valorization by Electrochemically Stimulated Mineralization from Mildly Acidic Conditions

Ivy Wu, Irene E.S. Walker, Robert T. Bell, Kerry C. Rippy*

National Renewable Energy Laboratory, 15013 Denver West Parkway, 80401 Golden, CO, USA

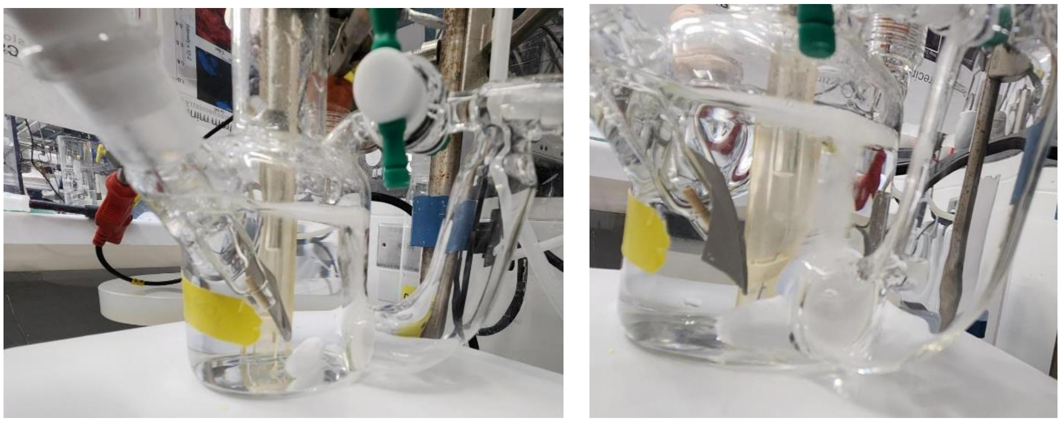

Figure S1. Photo of electrochemical cell with CO_2 bubbling, Ag/AgCl reference electrode, SS316 working electrode, and Pt mesh counter electrode. The surface area of the Pt mesh is >8x larger than the SS316.

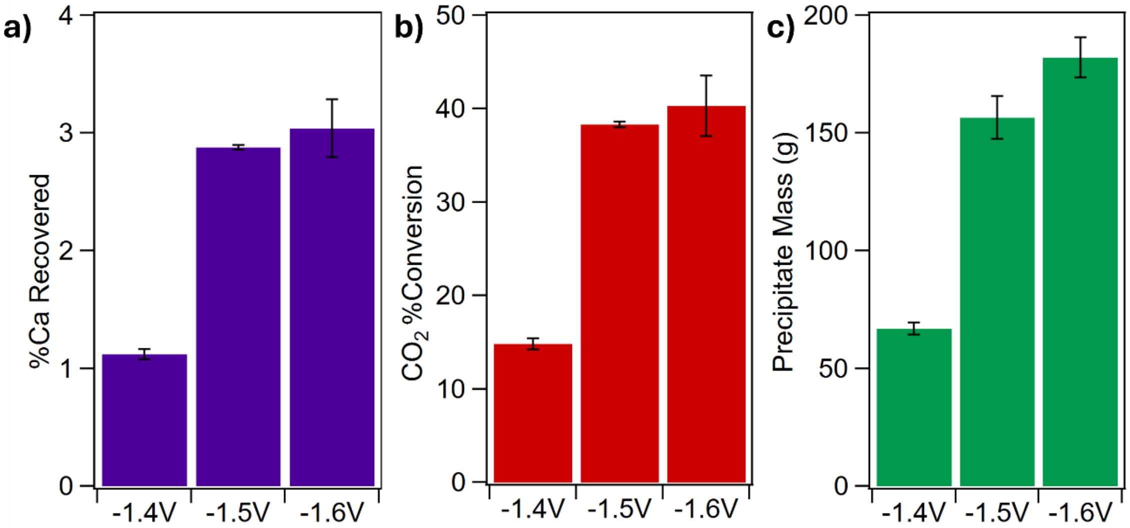

Figure S2. a) Ca recovery **b)** CO_2 Conversion to carbonate **c)** Total mass of precipitates formed at varying applied potentials on an initial surface area of 6.45 mm^2. CO_2 conversion was calculated with the assumption that the solution is saturated with CO_2 at the CO_2 solubility limit in water at room temperature[1]. Error bars indicate standard deviation of $n \geq 3$.

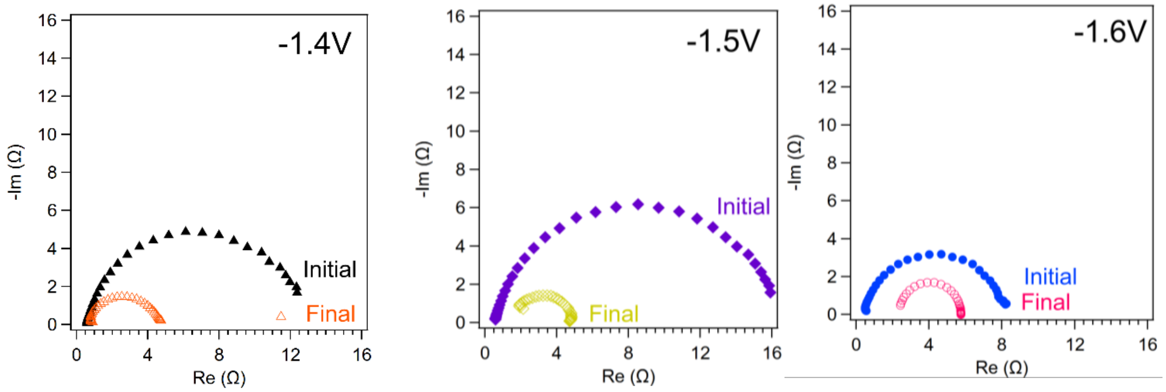

Figure S3. Nyquist plots showing the initial (filled markers) and final (open markers) condition at -1.4, -1.5,and -1.6 V.

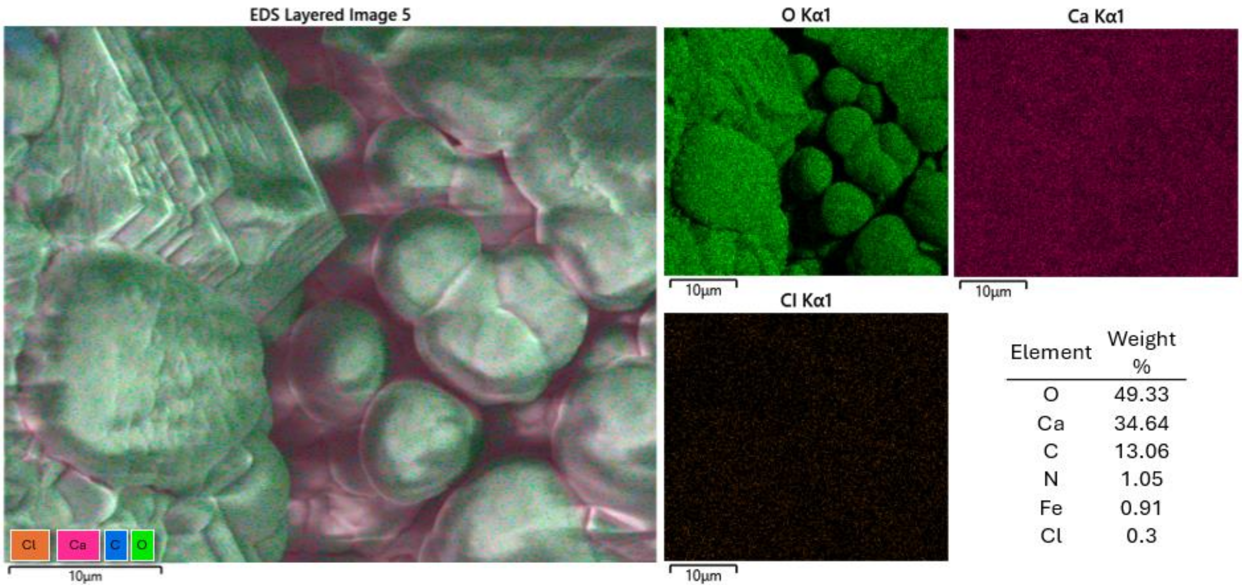

Element	Weight %
O	49.33
Ca	34.64
C	13.06
N	1.05
Fe	0.91
Cl	0.3

Figure S4. EDS mapping of precipitates formed from synthetic $CaCl_2$ solutions at -1.5 V chronoamperometry for 30 min.

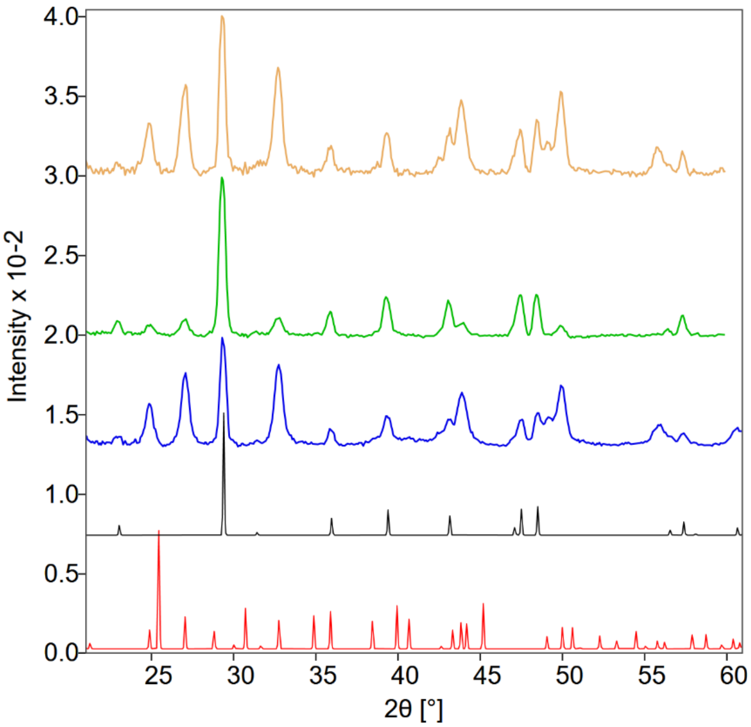

Figure S5. XRD pattern for precipitates formed from -1.4 V (orange), -1.5 V (green), -1.6 V (blue) vs Ag/AgCl, with reference peaks shown for calcite (black) and vaterite (red) from ICSD 52151, 18127-ICSD, respectively.

Table S1. Relative Standard Deviation (RSD) for efficiency and mass precipitated

Applied Potential (V)	Mol $CaCO_3$/mol e^- RSD%	Mass Precipitated RSD%
**-1.4**	10.61	7.91
**-1.5**	6.89	11.62
**-1.6**	10.19	9.23

References

1. Oloye, O. & O'Mullane, A. P. Electrochemical Capture and Storage of CO2 as Calcium Carbonate. *ChemSusChem* **14**, 1767–1775 (2021).
